# Risk of drug-induced cardiac arrhythmia during COVID-19 therapeutic treatment

**DOI:** 10.1186/s43044-021-00228-8

**Published:** 2021-11-18

**Authors:** Antonio Vitiello, Francesco Ferrara

**Affiliations:** 1 Pharmaceutical Department, Usl Umbria 1, Via XIV Settembre 06132, Perugia, Italy; 2 Pharmaceutical Department, Asl Napoli 3 Sud, Dell’amicizia street 22, 80035 Nola, Naples, Italy

**Keywords:** Arrhytmia, Cardiovascular, Cardiac risk, Pandemia, Virology

## Abstract

Therapeutic treatment of severe COVID-19 infection involves the administration of multiple pharmacologic agents to reduce the risk of serious complications; this may result in drug interactions and possible adverse reactions and induced cardiotoxicity. The risk–benefit ratio associated with the use of medications to treat COVID-19 should be carefully monitored. In addition, the severe COVID-19 patient may experience cardiac damage, and alteration of normal cardiac electrophysiology function. Severe COVID-19 with cardiac involvement and the risk of drug-induced adverse reactions may cause cardiac arrhythmias, including long qt syndrome, which in some cases may lead to sudden death. In this short review we briefly review the pharmacological agents used to treat severe COVID-19 with increased risk of causing long qt forms.

## Background

### Clinical aspects COVID-19

The COVID-19 viral epidemic that began in China in November 2019 has rapidly spread worldwide, transforming into a global pandemic in March 2020 [1]. To date, there are no direct antivirals against SARS-CoV-2 and therapeutic treatments are on an empirical basis, pharmacological agents such as antivirals, immunomodulators, anti-inflammatory agents, anticoagulants are used to prevent the most severe complications of infection [2–4]. SARS-CoV-2 penetrates cells via S protein through the angiotensin-converting enzyme-2 (ACE-2) receptor expressed on the cell surface, which is widely present in respiratory mucosal epithelial cells at the myocardial level [5] 6. In the most severe stages of infection, an irregular inflammatory cell infiltration composed of monocytes, macrophages, and lymphocytes infiltrate lung tissue inducing tissue damage [7]. In severe cases, the generalized inflammatory state is responsible for extrapulmonary injury, affecting other organs such as the heart or liver [8, 9]. Casual cardiac damage from COVID-19 includes pericarditis, acute coronary syndrome, electrophysiological disturbances, and the appearance of arrhythmias. A recent study [10] showed that 77% of deceased patients developed acute myocardial damage [11]. The molecular and pathophysiological basis suggests that the cause is attributable to the phenomenon of "cytokine storm" [12, 13] occurring in the most severe stages of COVID-19 infection, which may be the cause of myocarditis leading to acute heart failure and dysfunction of normal cardiac electrophysiology.

### COVID-19 and arrhythmias

Individuals with advanced age and cardiovascular disease are at increased risk for severe COVID-19 forms [14–16]. As described above, ACE-2 is the cellular entry receptor for SARS-CoV-2. ACE-2, in addition to the respiratory tract, is expressed in the cardiovascular system including the heart [17, 18]. The mechanisms of cardiac damage from COVID-19 are diverse, direct damage caused by the virus, and indirect damage caused by dysregulation of the ACE-2 signaling pathway [19] (studies show that ACE-2 levels decrease in severe stages of infection) and cytokine storm and subsequent induced myocarditis [20, 21]. Cardiovascular complications include malignant arrhythmias [22, 23]. Precautions and preventive measures are recommended, including electrocardiographic monitoring (ECG). Potential mechanisms that could lead to arrhythmogenesis among COVID-19 patients include hypoxia caused by direct involvement of viral tissue in the lungs, myocarditis, abnormal host immune response, myocardial ischemia, myocardial strain, electrolyte imbalances, intravascular volume imbalances, and drug side effects. In a study of 138 patients hospitalized with COVID-19, arrhythmia was detected in 17% of total patients and in 16 of 36 patients admitted to the ICU [24]. Therefore, a proarrhythmogenic effect of COVID-19 could potentially be related to disease outcome. Another study evaluated the risk of cardiac arrest and arrhythmias including atrial fibrillation (AF), bradyarrhythmias, and nonsustained ventricular tachycardia (NSVTs) in patients hospitalized for COVID-19. Among 700 patients, 9 cardiac arrests, 25 incident AF events, 9 bradyarrhythmias, and 10 NSVTs were identified. In addition, admission to the intensive care unit was associated with incident AF [25]. Another retrospective cohort study involved 1284 patients with severe COVID-19. In 44 of the 170 patients with cardiac injury (25.9%), ventricular tachycardia or fibrillation was detected [26]. Another study involved a total of 1197 health care providers surveyed. 5.6% of respondents identified presence of ventricular tachycardia/fibrillation in hospitalized COVID-19 patients. Amiodarone was the most commonly used antiarrhythmic drug for the management of ventricular arrhythmias [27]. Another epidemiological study showed that hospitalized patients who die from COVID-19 experience malignant cardiac arrhythmias more often than those who survive to discharge [28]. Cardiac arrests and arrhythmias are probably the consequence of systemic disease and adverse reactions to some drug treatments used and not only the direct effects of COVID-19 infection.

## Drugs used in covid-19 and risk of arrhythmias

The severe COVID-19 patient is treated with different pharmacological agents aimed at reducing the viral load, and the generalized inflammatory state [29], reducing the risk of thrombosis and avoiding serious injuries to vital organs such as lungs and heart [30–32]. Several pharmacological classes are used, off label antivirals, immunomodulators/anti-inflammatory agents, anticoagulants [32–34]. For these pharmacological treatments, the available evidence has shown results that are not always homogeneous and linear [35]. Recently, a massive vaccination campaign has started worldwide representing the most powerful weapon to stop the COVID-19 pandemic [36]. Some pharmacological agents used in COVID-19 patients may represent an additional risk of cardiotoxicity and induced cardiac electrophysiology dysfunction. In addition to this, some patients may be genetically predisposed to an increased risk of cardiac arrhythmias, such as long QT syndrome (LQTS) or Brugada syndrome (BrS). Hereditary factors, in addition to drug treatment, and severe COVID-19 infection, can represent a dangerous mix of proarrhythmogenic effects. To minimize the risk of cardiac arrhythmias in the severe COVID-19 patient, it is imperative to carefully monitor for potential Adverse Drug Reactions (ADRs) caused by drug-drug interactions, monitor the QT interval during drug therapy. Some evidence indicates that chloroquine/hydroxychloroquine might be useful in combating COVID-19 because of their antiviral potential [37], interfering with ACE-2 glycosylation and indirectly preventing endocellular penetration of SARS-CoV-2 [38, 39]. In some cases, the use of chloroquine has been associated with effects of QT prolongation and malignant arrhythmias. Indeed, chloroquine interacts with multiple cardiac ion channels, including the potassium channel of the human hERG gene; a reduction in the potassium current of the hERG channel is the main cause of drug-induced long QT syndrome. However, data on the increased risk of fatal arrhythmias in COVID-19 patients are conflicting. One study shows [40] that there was a small numerical excess of cardiac deaths (0.4%) but no difference in the incidence of new major cardiac arrhythmias among COVID-19 patients who received hydroxychloroquine compared with those who did not. Recent experimental data from a study recent have suggested that the QT prolongation effect of Clotokine in COVID-19 patients may be dose-dependent. Among 81 COVID-19 patients, 41 received high dose (i.e., 600 mg QC bid for 10 days) and 40 received low dose (450 mg bid on day 1 and qd for 4 days). The high-dose group presented more cases of QTc interval > 500 ms (18.9%) than the low-dose group (11.1%) [41]. Some in vitro evidence has shown that azithromycin possesses some antiviral activity and that it inhibits SARS-CoV-2 replication [42]. In addition to their in vitro antibacterial and antiviral activities, macrolides such as azithromycin are known to have immunomodulatory activity, reducing the production of pro-inflammatory cytokines and inhibiting neutrophil activation [43, 44]. However, epidemiologic data on the anti COVID-19 efficacy of azithromycin are counterintuitive. A large study of hospitalized COVID-119 patients showed that azithromycin did not improve survival or other prespecified clinical outcomes. The use of azithromycin in patients hospitalized with COVID-19 should be limited to patients in whom there is a clear antimicrobial indication [45]. Azithromycin may cause modest prolongation of the QT interval through increased cardiac Na + current and produce calcium overload in cardiocytes. Azithromycin can also cause ventricular tachycardia in the absence of QT prolongation [46]. However, epidemiologic data are unclear whether azithromycin as monotherapy used in COVID-19 patients increases the risk of malignant arrhythmias [47]. An antiviral directed against SARS-CoV-2 is not currently available; however, off-label antivirals licensed for other indications such as Remdesivir, lopinavir/Ritonavir have been used in this period. In vitro results demonstrate that even these antivirals show a risk of altering cardiac electrophysiology in a dose-dependent manner, and causing malignant arrhythmias [48]. The most severe stages of COVID-19 infection are characterized by a generalized hyperinflammatory state causing organ injury. In these stages the use of anti-inflammatory/immunomodulatory agents demonstrated some therapeutic efficacy. These agents include Tocilizumab, dexamethasone, colchicine, baricitinib [49–52]. Experimental data indicate a change in the duration of the Qtc interval following treatment with these pharmacological agents. Specifically evidence demonstrates that treatment with Tocilizumab, decreases the QTc interval [53]. In addition, a study serum kinase and glucocorticoid induction has been shown to increase human ether-a-gogo-related gene (hERG) protein expression levels and could mitigate QT prolongation [55, 56].

## Risk of arrhythmias induced by drug-drug and drug-pathology interactions

The therapeutic treatment of COVID-19 infection is complex, involving the administration of multiple pharmacological agents. Polypharmacy may increase the risk of drug-drug interaction, In addition, a possible drug-pathology interaction should also be considered. Chloroquine and hydroxychloroquine are metabolized by CYP3A4. Antiviral drugs used in COVID-19 infection, such as Remdesivir, Lopinavir/Ritonavir are potent enzymatic inhibitors of CYP3A4. The combination of chloroquine and antivirals may be associated with decreased chloroquine metabolization, increased plasma levels, and increased risk of QT prolongation [57, 58]. Therefore the QT interval should always be monitored, and if necessary administered a few hours apart. In addition, as described above, Remdesivir, lopinavir/ritonavir can also cause long QT, and in therapeutic combination with chloroquine/hydroxychloroquine increase the risk of malignant arrhythmias [58]. In addition, hydroxychloroquine/chloroquine is metabolized hepatically and excreted renally. In severe COVID-19 forms there may be hepatic and renal involvement with decreased function, in which case a dosage modification should be considered, to avoid elevated plasma concentrations of the drug and increased risk of QT long [59]. The use of azithromycin in combination with other drugs with risk of QT prolongation should be carefully monitored. In addition, dosage modification should also be considered for azithromycin in cases of decreased hepatic and renal function. The risk of drug-induced arrhythmias is much higher in critically ill COVID-19 patients. In addition, silent genetic long QT variants, found in approximately 4% of people, may make a person more vulnerable to malignant and fatal arrhythmias. Therefore, a large number of healthy individuals will be at an increased risk for drug-induced arrhythmias. Finally, other risk factors that may cause arrhythmias in the COVID-19 patient should be considered, for example, hypokalemia, profound hypoxemia, and cytokine storm [60]. Finally, little cardiac-safety data are available for the new monoclonal antibodies against COVID-19.

## Conclusions

Therapeutic treatment of COVID-19 infection may involve the administration of multiple pharmacologic agents, including chloroquine, hydroxychloroquine, antiviral drugs, macrolides, and monoclonal antibodies. Based on the available data, drugs such as chloroquine/hydroxychloroquine, remdesivir, and azithromycin may cause QT prolongation and cases of malignant arrhythmias. In addition, COVID-19 disease, particularly if there is cardiac involvement, and underlying pathologies may increase the risk of arrhythmias. Careful monitoring of drug therapies should always be performed, and clear administration protocols should be in place in every hospital and clinic using these drugs to treat COVID-19.

## Data Availability

Full availability of data and materials.

## Abbreviations

ACE-2: Angiotensin-converting enzyme-2
NSVTs: Nonsustained ventricular tachycardia
AF: Atrial fibrillation
ECG: Electrocardiographic monitoring
LQTS: Long QT syndrome
BrS: Brugada syndrome
ADRs: Adverse Drug Reactions
hERG: Human ether-a-gogo-related gene
